# A novel homozygous *SLC19A2* mutation in a Portuguese patient with diabetes mellitus and thiamine-responsive megaloblastic anaemia

**DOI:** 10.1186/s13633-015-0002-6

**Published:** 2015-04-15

**Authors:** Sophia Tahir, Lieve GJ Leijssen, Maha Sherif, Carla Pereira, Anabela Morais, Khalid Hussain

**Affiliations:** Developmental Endocrinology Research Group, Clinical and Molecular Genetics Unit, Institute of Child Health, University College London, 30 Guilford Street, London, WC1N 1EH UK; Endocrinology Unit, Department of Paediatrics, Hospital de Santa Maria – CHLN, Lisbon, Portugal; Hematology Unit, Department of Paediatrics, Hospital de Santa Maria – CHLN, Lisbon, Portugal; Department of Paediatric Endocrinology, Great Ormond Street Hospital for Children, NHS Foundation Trust, WC1N 3JH London, United Kingdom

**Keywords:** TRMA, Diabetes mellitus, Sensorineural deafness, Megaloblastic anaemia, *SLC19A2* mutation

## Abstract

Thiamine-responsive megaloblastic anaemia (TRMA) is a rare syndrome where patients present with early onset diabetes mellitus, megaloblastic anaemia and sensorineural deafness. This report describes a new case of TRMA syndrome in a female patient of Portuguese descent, born to unrelated parents. The patient was found to have a novel homozygous change R397X in exon 4 of the *SLC19A2* gene, leading to a premature stop codon. The patient’s diabetes and anaemia showed a good response to daily thiamine doses, reducing the daily insulin dose requirement. The report further indicates that TRMA is not only limited to consanguineous or ethnically isolated families, and should be considered as a differential diagnosis for patients presenting with suggestive clinical symptoms.

## Introduction

Thiamine-responsive megaloblastic anaemia (TRMA; OMIM 249270), also called Roger’s syndrome, is a very rare autosomal recessive disorder characterized by early onset diabetes mellitus (DM), megaloblastic anaemia and sensorineural deafness. The syndrome was first described by Rogers et al. [1]. Other reported clinical findings in addition to the characteristic triad include congenital heart disease, arrhythmias, retinal degeneration, optic atrophy, aminoaciduria, short stature, situs inversus, Polycystic Ovarian Syndrome and stroke [2]. The disease can manifest anytime between infancy and adolescence, although often not all of the cardinal findings are manifested at onset. The variable phenotypic presentation of TRMA syndrome may cause a significant delay between the onset of symptoms and an accurate diagnosis. Treatment is symptomatic and includes pharmacological doses of thiamine (vitamin B1), which usually corrects hematological and endocrine function, but neurological manifestations do not respond as well [1,3].

Most of the TRMA patients originated from consanguineous families, which is consistent with autosomal recessive inheritance [4]. TRMA is caused by homozygous mutations in the *SLC19A2* gene [4], which encodes a high-affinity thiamine transporter 1 protein (THTR-1) [5,6], located on chromosome 1q23.3. The human TRMA gene contains six exons that encode a protein of 497 amino acids with 12 transmembrane domains [7]. *SLC19A2* is expressed in a wide range of human tissues including bone marrow, liver, colon, small intestine, pancreas, brain, retina, heart, skeletal muscle, kidney, lung, placenta, lymphocytes and fibroblasts [8].

To date, TRMA syndrome has been reported in around 30 families worldwide. Its prevalence and incidence are unknown. At least 43 mutations in the *SLC19A2* gene have been found to cause TRMA. Most of these mutations lead to the production of an abnormally short, non-functional THTR-1. Other mutations change single protein building blocks (amino acids) in THTR-1, which disrupts the proper folding of the protein or prevents it from reaching the cell surface. All of these mutations prevent THTR-1 from bringing thiamine into the cell.

Here we report a female patient with TRMA, belonging to a non-consanguineous family of Portuguese descent. A novel homozygous mutation was found in the *SLC19A2* gene leading to a premature stop codon. The patient presented with the typical features TRMA, diabetes mellitus, sensorineural deafness and megaloblastic anaemia.

## Case presentation

A girl, 14 months of age, was born at full term, birth weight 3.03 kg after an uneventful pregnancy and delivery to non-consanguineous parents of Portuguese descent. Her mother is known with thalassemia minor, her father is healthy. A second degree paternal cousin is known with DM type 1, diagnosed at the age of 31 years.

At the age of 10 months, our patient was referred to the hospital because of fever of unknown origin. At that time, she was found to have hyperglycaemia of 509 mg/dl [28 mmol/L] and diabetic ketoacidosis (pH 7.26, HCO_3_ 10.9, serum and urine ketones ++). Other laboratory evaluation revealed anaemia (Hb 8.3 g/dl) and high CRP (17.1 mg/dl). Cardiovascular examination showed an I/VI systolic heart murmur and she presented with skin pallor. Although treatment with insulin was started according to the diabetic ketoacidosis protocol, there was progressive Hb lowering (6.5 g/dl on day 10), a reticulocyte count of 54%, low neutrophil (600 with WBC 10.700/mm^3^) and low platelets (29000/mm^3^). For this, she was treated with a blood transfusion at that time.

In the following months there was still progressive Hb lowering, maintaining MCV (81-87 fL), leukocytes between 7500 and 11900 with neutrophils <3000 and platelets went down to 67000/mm^3^. In addition, she needed another transfusion during febrile illness, due to CMV infection.

At 13 months of age she had ENT evaluation and hearing evoked potentials were made, confirming sensorineural deafness, which is treated with hearing prosthesis. In the meantime, she needed progressive growing doses of insulin (at 14 months 0.75 U/kg).

After institution of thiamine supplementation (75 mg/day) and another blood transfusion, due to a rhinopharyngitis (Hb 6.4 g/dl and platelets 101000/mm^3^), the haemoglobin (present: 11.5-12.9 g/dl) and WBC (present: 12000-14000/mm^3^ with neutrophils 4.200-4.680/microlL, platelets >500000/mm3) increased. In addition, the patient’s blood glucose showed an improvement (HbA1c: 6.5%, 47.5 mmol/mol) and her daily insulin requirements progressively decreased to a 2U/day (0.15 U/kg/day).

**Figure 1:**
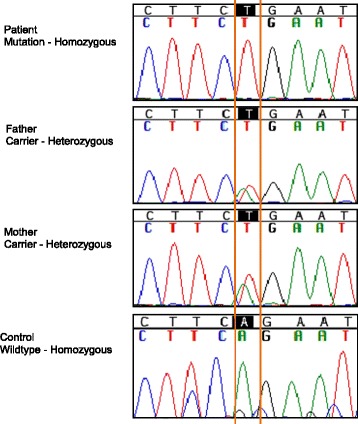
***SLC19A2*** **Mutation analysis: Chromatograms showing the location of the c.1189A>T/R397X mutation in our patient, her parents and a control.** The patient is homozygous (T/T) for the mutation, whilst both parents are heterozygous (A/T) carriers. The wild-type allele at same location is a homozygous (A/A) as shown in the control.

Considering the clinical history of the patient, mutational analysis of the *SLC19A2* gene was conducted. Primers were designed to cover all six exons and their flanking intronic regions using the online Primer 3 software [9]. The sequencing reaction was conducted using the BigDye Terminator V1.1 Cycle Sequencing kit (Applied BioSystems, Foster City, CA, USA). The patient was found to be homozygous for the c.1189A>T nucleotide change (protein change R397X) in exon 4 of the *SLC19A2* gene. This mutation was absent from dbSNP database [10], 1000 Genomes Project [11] and in Exome Variant Server [12] in the homozygous state. The mutation leads to stop codon and causes an early protein truncation and was predicted to be deleterious by the online PROVEAN PROTEIN software [13]. The c.1189A>T change is previously reported (SNP ID: rs371383730) in the heterozygous state in a single European-American individual on the Exome Variant Server [12]. The parents of the patient were also found to be heterozygous carriers for the mutation, which was absent in the control (Figure 1).

## Discussion

The first case of TRMA syndrome was described in 1969 by Rogers et al. [1]. Most of the early cases which followed have been in patients from a Middle Eastern or Asian background. However a growing number of cases recently report patients from non-consanguineous European backgrounds [2,14,15]. Here we report a female patient from a non-consanguineous Portuguese family with a homozygous nonsense mutation in the *SLC19A2* gene.

Thiamine plays an essential role in carbohydrate metabolism and energy production reactions. Under physiological concentrations of thiamine as obtained from normal diet, it is transported by two high affinity carriers found in the intestine - THTR-1 encoded by the *SLC19A2* gene, and THTR-2 encoded by the SLC19A3 gene. Both genes are expressed in most body tissues; however the cochlear inner hair cells, pancreatic islet cells and erythropoietic precursor cells only express *SLC19A2*. Under high thiamine concentrations, thiamine crosses the cell membrane through passive diffusion [8,16-18]. In TRMA syndrome the active uptake of thiamine by carrier mediated mechanisms is disrupted leading to thiamine deficiency. As most cells have a compensatory mechanism via THTR-2, only the three THTR-1 dependent cells are the most affected by thiamine deficiency, and thus explain the clinical triad associated with TRMA syndrome [19].

The high level of thiamine in pancreas is essential for its normal endocrine and exocrine function [20]. Thiamine deficiency negatively impacts insulin regulation, as the pancreatic islet cells from thiamine deficient rats show impaired insulin secretion [21]. This is possibly due to cell death and apoptosis [22].

Thiamine is essential for normal auditory development and function, and infants fed thiamine deficient meals develop auditory problems [23]. The higher expression of THTR-1 in the cochlear inner hair cells than the outer hair cells suggests its essential role for inner hair cell function [24]. Slc19a2 null mice show selective loss of inner hair cells, within 1-2 weeks of being subjected to thiamine deficient diet [25].

To date 43 mutations have been described in the *SLC19A2* gene (HGMD January 2015) [26]. Majority are missense and nonsense changes, followed by small insertions and deletions. A large intragenic deletion was also reported by Beshlawi et al., where a 5224 base pair region in *SLC19A2* was deleted in 6 patients [27]. Cases reporting compound heterozygosity show that TRMA is not only limited to consanguineous or ethnically isolated families [2,14,15].

Most TRMA patients show the cardinal findings of the syndrome; however the onset of the different symptoms may vary [2]. Our patient developed all 3 symptoms between 10 to 13 months of age. Furthermore the observation of other clinical symptoms in TRMA apart from the clinical triad makes it difficult to define a clear genotype-phenotype relationship. Visual impairments, cardiac anomalies and neurological impairments are seen in some patient with TRMA and not others, despite carrying the same causative mutation [28-30].

Treatment with high thiamine doses produces a variable response in patients [31]. Usually this corrects for haematological and endocrinal findings, normalizing patient’s anaemia and hyperglycaemia overtime. However patients have been reported where insulin therapy was still required [31]. Thiamine supplementation becomes ineffective once the patient reaches puberty; follow up studies report patients requiring insulin therapy and regular blood transfusion during adulthood [7]. Low thiamine concentrations may cause cell death by apoptosis in TRMA patient fibroblast cells [22]. This may therefore lead to a reduced reserve of pancreatic beta cells, and as suggested by Valerio and colleagues, may be insufficient to meet the changing physiological demands of insulin during puberty [32]. Our patient required progressively increasing doses of insulin to control her blood glucose levels prior to TRMA diagnosis. This was reduced to a single dose of 2U/day (0.15U/kg/day) of insulin within 6 months of beginning on a thiamine dose of 75 mg/day.

Hearing loss in *SLC19A2* knockout mice can be reversed when fed with a high thiamine diet following a thiamine free diet [33]. However in humans the effect of thiamine deficiency seems to be irreversible for hearing loss [31]. One TRMA patient diagnosed without the development of hearing loss at one month of age, was started on early thiamine therapy, and still had no signs of hearing loss in the follow up period at 30 months of age [34]. Early intervention in this case could have occurred before the damage of thiamine deficiency affected hearing. It may still be possible that the patient goes on to develop hearing loss later in life. Patients with compound heterozygote mutations are reported to have a milder phenotype, with less severe hearing loss [2].

## Conclusion

In summary we report a new case of TRMA syndrome in a patient from a non-consanguineous European family of Portuguese descent. The causative mutation, c.1189A>T/ R397X is a novel nonsense mutation, introducing a premature stop codon in exon 4 for the *SLC19A2* gene. Increasing number of cases are reported in European non consanguineous families, and therefore despite being rare, TRMA should be considered as a differential diagnosis in patients presenting with suggestive clinical symptoms. The patients generally respond well to thiamine supplementation, which keeps the condition under control – although only temporarily till the patient reaches puberty.

## Consent

Written informed consent was obtained from the patient for publication of this Case report and any accompanying images. A copy of the written consent is available for review by the Editor-in-Chief of this journal.

## Abbreviations

DM: Diabetes mellitus
TRMA: Thiamine responsive megaloblastic anaemia
THTR-1: High-affinity thiamine transporter 1 protein
THTR-2: High-affinity thiamine transporter 2 protein
SLC19A2: Solute carrier family 19 (thiamine transporter), member 2
SLC19A3: Solute carrier family 19 (thiamine transporter), member 3
